# Identification of an immune classification for cervical cancer and integrative analysis of multiomics data

**DOI:** 10.1186/s12967-021-02845-y

**Published:** 2021-05-10

**Authors:** Xintong Lyu, Guang Li, Qiao Qiao

**Affiliations:** Department of Radiation Oncology, First Hospital of China Medical University, Heping District, Shenyang,, Liaoning China

**Keywords:** Immune classification, Cervical cancer, Multiomics

## Abstract

**Background:**

To understand the molecular mechanisms of the antitumour response, we analysed the immune landscape of cervical cancer to identify novel immune molecular classes.

**Methods:**

We established a stable immune molecular classification using a nonnegative matrix factorization algorithm and validated the correlation in two validation sets of 249 samples.

**Results:**

Approximately 78% of cervical cancers (CCs) (228/293) were identified to show significant enrichment in immune cells (e.g., CD8 T cells and macrophages), a type I IFN response, enhanced cytolytic activity and high PDCD1, and these CCs were referred to as the “immune class”. We further identified two subtypes of the immune class: active immune and exhausted subtypes. Although the active immune subtype was characterized by enrichment of IFN signatures and better survival, the exhausted subtype expressed activated stroma, a wound healing signature, enhanced M2 macrophages and absence of CD8 T cells and the TGF-β response signature. Integrative analysis of multiomics data identified EGFR, JUN, MYC, FN1 and SERPINE1 as key modulators of the tumour immune microenvironment and potential targets for combination therapies which was validated in two validation sets.

**Conclusions:**

Our study introduces a novel immune classification that might predict ideal candidates to receive immunotherapy or specific combination therapies.

**Supplementary Information:**

The online version contains supplementary material available at 10.1186/s12967-021-02845-y.

## Introduction

Cervical cancer (CC) remains the fourth most commonly diagnosed female malignancy and one of the leading causes of cancer-related mortality worldwide, especially in developing countries [1, 2]. More than half of all CC cases are currently diagnosed at advanced stages [3]. For patients with recurrent and metastatic cancers, therapeutic options are extremely limited. Chemotherapy doublets combined with bevacizumab, constitute first-line therapy for recurrent and metastatic cervical carcer. Although the survival was improved, bevacizumab is not curing anyone, and while GOG 240 addressed an unmet clinical need in a high-risk population who progress on first-line therapy. Progress definitely, but much more is required [4].

In recent years, immune checkpoint inhibitors (ICIs) that initiate effective antitumour activity have driven further exploration of this new therapy in CCs. In June 2018, pembrolizumab was approved for the treatment of patients with recurrent or metastatic CC with disease progression on or after chemotherapy whose tumours express PD-L1 in ≥ 1% of cells by immunohistochemistry. Despite the promising anticancer activity, only a fraction of patients exhibited dramatic effects on single-agent anti-PD-L1/PD-1 antibody treatment. In the KEYNOTE-158 study, patients were treated with pembrolizumab, the median follow-up was 10.2 months, and the objective response rate (ORR) was only approximately 12.2% [5]. To determine the population that will benefit from PD-1 blockade treatment, there are 4 FDA-approved assays of PD-L1 expression by immunohistochemistry to help guide decisions. However, PD-L1 expression before immunotherapy may be useful but not sufficient to act as a definitive predictive biomarker. For instance, patients with melanoma can exhibit a clinical response regardless of PD-L1 expression status.

Successful antitumour immune responses following PD-1/PD-L1 blockade require reactivation and clonal proliferation of tumour-specific T cells in the tumour microenvironment (TME), and the differences in the effects of ICIs can be partially attributed to the heterogeneity of the TME [6, 7]. The “hot”, immune-inflamed TME that is associated with higher densities of CD8 + tumour-infiltrating lymphocytes (TILs) may predict benefit from anti-PD-1 therapy. In contrast, non-inflamed tumours with a “cold” TME generally respond poorly to anti-PD-L1/PD-1 therapy [8–10]. Estimation of non-cancerous cell proportions from samples can be performed using genomics data. During the last decade, multiple computational approaches have been developed intending to calculate TME cell type population estimates and we adopt several of them for TME analysis [11].

However, why some tumours are “inflamed” with effector T cell infiltration whereas others are not remained to be elucidated. The elicited durable clinical responses to PD-1 pathway blockade largely depend on TME profiles. Because of the complexity of TME, combination therapies represent the next wave of clinical cancer treatment to overcome the limitations associated with single-agent therapy [12]. Therefore, for rational combination therapies, we aim to provide targets in various TME subtypes and an integrative stable immune class that may predict benefit from single agents or combination therapies in specific patients.

As we all know, tumours are complex mixtures of stromal cellular elements. Nonnegative matrix factorization (NMF) is a virtual separation approach that could help separate molecular signatures of tissue compartments from measurements of bulk tumor samples. It is well suited for biological data as it constrains all sources to be positive in nature. Moffitt RA et al. have recently demonstrated that NMF is useful for analyzing gene expression to identify tumour-specific and stroma-specific subtypes with biologic relevance in pancreatic ductal adenocarcinoma [13].

Using NMF, we deconvoluted gene expression data and isolated the inflammatory signal to characterize the immunologic landscape of CC. We identified an immune-specific class of CC associated with prognosis and immune modulatory alterations and conducted an integrative analysis of multiomics data to identify key modulators of the tumour immune microenvironment and potential individual treatments in various immune classes.

## Materials and methods

### Patients and samples

For the purpose of the study, the gene expression profiles from a total of 542 human cervical cancer samples were analysed (Additional file 1: Fig. S1). All samples of the training set were previously obtained from TCGA. RNA profiling, CNV data and mutation data were available for all 291 samples. An additional 249 samples of patients from 2 datasets of GEO (GSE63514 and GSE68339) were used for external validation [14, 15].

### Identification of the immune class

Virtual microdissection of gene expression data was performed in the training set using unsupervised NMF [16], as previously described [13], with k = 3 as the number of factors. An immune-related expression pattern was revealed by integrating NMF-identified factors with the immune enrichment score calculated by single-sample gene set enrichment analysis (ssGSEA). Once the immune expression pattern was deconvoluted by NMF and characterized by integration with ssGSEA scores, we listed the top-ranked genes according to their weighting. Unsupervised clustering of the top-ranked genes was then performed.

### Molecular characterization of the immune class

Enriched molecular pathways and gene expression signatures were evaluated using GSEA and ssGSEA. Previously published cervical cancer molecular classifications were analysed [17–19]. We identified differentially expressed genes (DEGs) between immune class and non-immune class with criteria of false discovery rate < 0.05 and log fold-change > 1.0 by the R package “limma”. Gene set enrichment analysis (GSEA) was applied to identify pathways enriched in each subgroup and was performed on a Java GSEA desktop application (www.broad.mit.edu/gsea/). Absolute immune cell scores from gene expression datasets were computed for 22 immune cell types according to gene signatures from CIBERSORT (https://cibersort.stanford.edu/) [20].

### Genomic correlations with the immune class

Scores for copy number burden, aneuploidy, homologous recombination deficiency (HRD), SNV neoantigens, and non-silent mutation rate were derived [18]. The number of altered fractions in the copy number burden score and the number of segments represented the fraction of bases deviating from baseline ploidy (defined as above 0.1 or below—0.1 in the log2-normalized relative copy number (CN) space) and the total number of segments in each copy number profile, respectively. Altered aneuploidy scores were calculated as the sum of amplified or deleted arms [21]. In addition, Spearman correlations were determined between the leukocyte fraction and measures of DNA alteration.

### Copy number variation analysis

The TCGA copy number (gene-level) data were downloaded from https://genome-cancer.ucsc.edu/ in January 2020. Segment mean values larger than 0.3 were defined as copy number gains, and those less than -0.3 were defined as copy number losses. The chi-square test was adopted to identify the significantly different copy number variants (CNVs) between immune subgroups, and Circos analyses were performed by the R package “Rciorcos” [22].

### Somatic mutation analysis

Somatic mutation profiles, which are available from the Genomic Data Commons Data Portal (https://portal.gdc.cancer.gov/), detected by VarScan 2 and with a somatic mutation frequency > 5% were considered to compare values among distinct subgroups. OncoPrints for somatic mutation patterns were generated by the R package “maftools”. Michael R. Stratton reported a mathematical approach and computational framework to extract mutational signatures from catalogues of somatic mutations from cancers and identified 33 mutational signatures in all cancer types [23].

### MicroRNA expression and long noncoding RNA expression analysis

The lncRNA and miRNA data were downloaded from https://genome-cancer.ucsc.edu/. Among the candidate miRNAs and lncRNAs, we further identified potential regulators and target genes using the following criteria: the mature miRNA has experimentally validated targets from miRTarBase [24] and computationally predicted targets from two well-established miRNA target prediction databases, miRanda and miRDB [25, 26]. For differentially expressed functional miRNAs (DEFMs) analysis, we used miRNA data in limma with adjusted P-value of ≤ 0.05. In addition, we identified differentially expressed lncRNAs between immune class and non-immune class with criteria of false discovery rate < 0.05 and log fold-change > 1.0 by the R package “limma”.

### Protein expression analysis

Three subgroups were used to perform comparisons using protein (RPPA) data from https://www.tcpaportal.org/tcpa/download.html. Ranking with the t-test p-value generated the top-ranked proteins induced in the subgroups, using a cut off t-test p-value of < 0.05. T-test were performed using the R language. The Search Tool for the Retrieval of Interacting Genes (STRING) database was used to construct a protein–protein interaction network of top-ranked proteins.

### Validation in independent datasets

The immune class was generated using the “NMF method” in the training set (n = 293). The ability of the “NMF method” to capture the immune class was validated in our validation sets (GSE63514 and GSE68339). In addition, GSEA was applied to validate pathways enriched in the immune class.

### Statistics

Statistical analyses were performed with SPSS Statistics software version 24 and R software 3.6.1. Correlations between immune classes and clinicopathological variables were analyzed by Chi-square test (and the Fisher's exact test when appropriate) and Wilcoxon rank-sum test for categorical and continuous data, respectively. Correlations between immune classes and three published molecular classes were analyzed by Chi-square test. One-way ANOVA and Least—Significant Difference (LSD) tests were performed for multiple group comparisons. Kaplan–Meier estimates and log-rank test were performed to analyze the association of immune classes with overall survival. The propensity score matching (PSM) was performed for adjustment for risk factors. P-values < 0.05 were considered statistically significant.

## Results

### A novel molecular immune class of cervical cancer

We first performed NMF in the training cohort (n = 293) to extract gene expression signatures related to immune pathways (Additional file 1: Fig. S1). We confirmed that one of the NMF-identified clusters was linked to inflammatory markers and immune cells, which was corroborated by an observed lowest immune enrichment score, as previously reported [8, 10] (Additional file 1: Fig. S2). Consensus clustering of exemplar genes identified a new molecular immune phenotype present in 77.82% of the cohort (228/293), which we refer to herein as the “immune class” (Additional file 1: Fig. S3). We refer to the other 22.18% of the cohort herein as the “non-immune class”. Samples within the immune class showed significant enrichment of signatures identifying immune cells (e.g., CD8 T cells, B cells, DCs, and macrophages), the type I IFN response, and enhanced cytolytic activity (ANOVA, all, P < 0.001) (Additional file 1: Fig. S3). In contrast, we detected strong and significant lack of signatures identifying immune cells and lower immune enrichment score in the non-immune class (ANOVA, all, P < 0.001). The class comparison identified 1,497 genes that were significantly upregulated in the immune versus non-immune classes (Additional file 1: Table S1). GSEA identified TNF alpha signalling via NF-KB and IFN-related signalling in immune class, while oxidative phosphorylation and xenobiotic metabolism pathways in non-immune class (FDR < 0.05, Additional file 1: Fig. S4, Table S2).

### Two subtypes of the tumour microenvironment in the immune class: active immune and exhausted classes

**Fig. 1:**
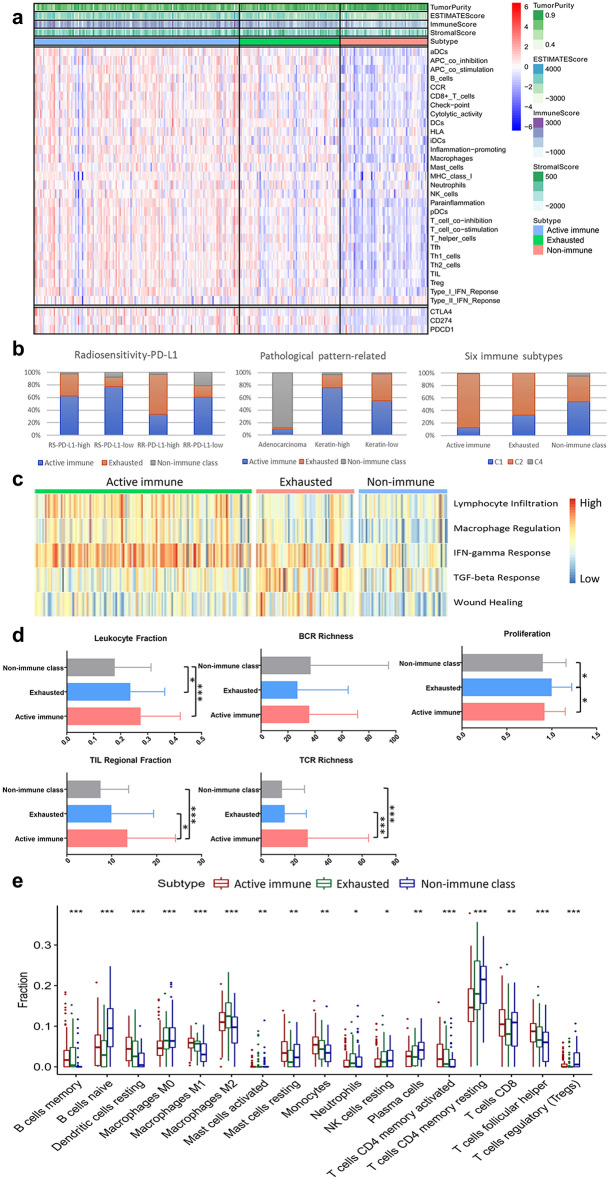
Identification of the immune class of CC and the molecular characterization of the subgroups. **a** NMF analysis of whole-tumour gene expression data using a molecular signature able to identify the immune class of CC. In the heatmap, high and low gene set enrichment scores are represented in red and blue, respectively; the same representation is used for high and low gene expression. **b** An integrated analysis of these immune molecular subgroups with the three published molecular classes. **c** The five modules of the immune subgroups are indicated by the heatmap. High and low scores are represented in red and blue, respectively. **d** Correlation of key immune characteristics with immune subgroups. **e** Infiltration of immune cells by immune subgroups. *p < 0.05; **p < 0.01; ***p < 0.001

Given that the immune system exerts both antitumour and protumour activity, we next explored the type of immune modulation in response to the tumour microenvironment within the immune class. Figure 1a shows that 32.89% of samples in the immune class (75/228) were characterized by a previously reported activated stromal gene signature that captures the activated inflammatory stromal response [13]. Samples with the activated stromal gene signature were associated with a lower immune enrichment score (ANOVA, P = 0.002) than samples lacking the activated stroma signature. We named these two clusters exhausted and active immune subgroups. Clinicopathological data and follow-up data for patients included in the training datasets are summarized in Table 1. Among these immune subgroups, the significantly different factors include HPV status (ANOVA, P = 0.002), HPV16/18 state (ANOVA, P < 0.001) and pathological type (ANOVA, P < 0.001). We found that patients negative for HPV were most abundant in the non-immune class, whereas patients with negative for HPV16/18 state were most common in the exhausted subgroup. In addition, the non-immune class consisted mostly of adenocarcinomas compared with other subgroups. Including these factors, univariate and multivariate analysis were performed (Additional file 1: Tables S3, S4). We also observed enrichment of PDCD1 in the active immune subgroup, which was previously reported to predict anti-PD1 treatment response (ANOVA, P < 0.001) [27]. Samples in the exhausted subgroup showed significant enrichment of signatures identifying immune cells (e.g., macrophages), an activated stroma, and the TGF-β response signature (ANOVA, all, P < 0.001). DEGs between the classes are shown in Additional file 1: Table S5. GSEA confirmed the driver role of the oxidative phosphorylation pathway, as well as myc target pathways, in the exhausted subgroup (FDR < 0.05, Additional file 1: Fig. S5, Table S6).

**Table 1:** Clinicopathological characterization of patients included in the training datasets

Variable	Group			P value
	Active immune (n = 153)	Exhausted (n = 74)	Non-immune (n = 64)	
Age	48.86 (14.29)	47.31 (14.00)	47.17 (12.43)	0.611
BMI	28.54 (8.52)	27.60 (6.32)	26.92 (5.97)	0.366
Smoke				0.852
Yes	32 (20.9%)	19 (25.7%)	12 (18.8%)	
No	101 (66.0%)	46 (62.2%)	45 (70.3%)	
Unknown	20 (13.1%)	9 (12.2%)	7 (10.9%)	
HPV state				0.002
Positive	147 (96.1%)	70 (94.6%)	53 (82.8%)	
Negative	6 (3.9%)	4 (5.4%)	11 (17.2%)	
HPV16/18 state				< 0.001
Positive	107 (69.9%)	42 (56.8%)	39 (60.9%)	
Negative	45 (29.4%)	32 (43.2%)	13 (20.3%)	
Unknown	1 (0.7%)	0 (0.0%)	12 (18.8%)	
Clinical stage				0.121
Stage I	84 (54.9%)	32 (43.2%)	43 (67.2%)	
Stage II	35 (22.9%)	18 (24.3%)	11 (17.2%)	
Stage III	23 (15.0%)	13 (17.6%)	5 (7.8%)	
Stage IV	7 (4.6%)	9 (12.2%)	5 (7.8%)	
Unknown	4 (2.6%)	2 (2.7%)	0 (0%)	
Lymph node state				0.289
Yes	95 (62.1%)	37 (50.0%)	42 (65.6%)	
No	22 (14.4%)	11 (14.9%)	8 (12.5%)	
Unknown	36 (23.5%)	26 (35.1%)	14 (21.9%)	
Radiation therapy				0.931
Yes	77 (50.3%)	35 (47.3%)	31 (48.4%)	
No	31 (20.3%)	13 (17.6%)	12 (18.8%)	
Unknown	45 (29.4%)	26 (35.1%)	21 (32.8%)	
Pathological type				< 0.001
Squamous	150 (98.0%)	73 (98.6%)	18 (28.1%)	
Adenosquamous	0 (0.0%)	1 (1.4%)	3 (4.7%)	
Adenocarcinoma	3 (2.0%)	0 (0.0%)	43 (67.2%)	

To explore the relationship of this immune-related classification of CC and signatures capturing the presence of immune cells and IFN-γ-signaling, the pathological pattern and the response to radiation and immune checkpoint therapy. We next sought to integrate these immune molecular subgroups with the three published molecular classes in cervical cancer [17–19]. For Radiosensitivity-PD-L1 classification [19], we detected the highest proportion of the active immune subgroup within the radiosensitive (RS)-PD-L1-low subtypes (Chi-square, 77.5%, P < 0.05), while the exhausted immune subgroup of tumours harboured the highest proportion of the radioresistant (RR)-PD-L1-high subtype (Chi-square, 63.3%, P < 0.05). These observations are in keeping with a report from Tobin Strom et al. suggesting that upon tumour generation, the immune system was activated and was associated with high radiosensitivity [28]. We also found the highest frequency of the non-immune class in the RR-PD-L1-low subtypes (Chi-square, 21.2%, P < 0.05) (Fig. 1b). For the pathological pattern-related classification [17], we found that approximately 90% of non-immune samples belonged to the adenocarcinoma subtype, while exhausted samples accounted for 52.5% of keratin-low samples (Fig. 1b). In line with previous data, the results suggested that active immune response were more frequently distributed in squamous cell carcinoma than those in adenocarcinoma [29]. For integration with the six pan-cancer immune subtypes [18], we found that approximately 97% of CC patients belong to the wound healing (C1) (26.3%) or IFN-γ-dominant (C2) (70.6%) subtypes, while other subtypes accounted for only 3% of CC samples (Fig. 1b) [30]. It is suggested that the IFN-γ-dominant subtype could benefit from immunotherapy, and as expected, the highest proportion of the active immune subgroup (85.6%) was shown in the IFN-γ-dominant subtype. All things considered, these analyses suggest that we successfully identified an immune-related class of CC enriched with signatures capturing radiosensitivity and PD-L1 expression, signatures of pathological pattern and signatures of response to immunotherapy.

### Molecular characterization of the immune class

Additionally, the active immune subgroup was significantly associated with lymphocyte infiltration, macrophage regulation and IFN-γ response, while the exhausted subgroup showed significant correlations with immunosuppressive components, e.g., the TGF-β response signature and the wound healing signature, which suggest fibroblasts activation [31] (Fig. 1c). In addition, the highest TIL proportions and TCR expression were detected in patients within the active immune subgroup (ANOVA and LSD test, P < 0.05) (Fig. 1d). Conversely, in samples within the exhausted subgroup, we observed the highest proliferation rate (ANOVA and LSD test, P < 0.05) which may suggest tumour-promoting characteristics (Fig. 1d). In contract, the non-immune class showed lowest immune activating component including lymphocyte infiltration, macrophage regulation and IFN-γ response. To further explore infiltrating immune cells in detail, we analysed the type of infiltrating immune cells in samples within the three subgroups. Among these cell populations, memory B cells, resting dendritic cells (DCs), M1 macrophages, resting mast cells, monocytes, activated memory CD4 T cells and helper follicular T cells were significantly increased in the active immune subgroup. Tumour samples in the exhausted subgroup showed the highest infiltration of M2 macrophages, M0 macrophages and neutrophils. The non-immune class had the highest density of naïve B cells, plasma cells, resting memory CD4 T cells, CD8 T cells and regulatory T cells (Tregs) (ANOVA and LSD test, P < 0.05) (Fig. 1e).

### Validation of the immune class across datasets

**Fig. 2:**
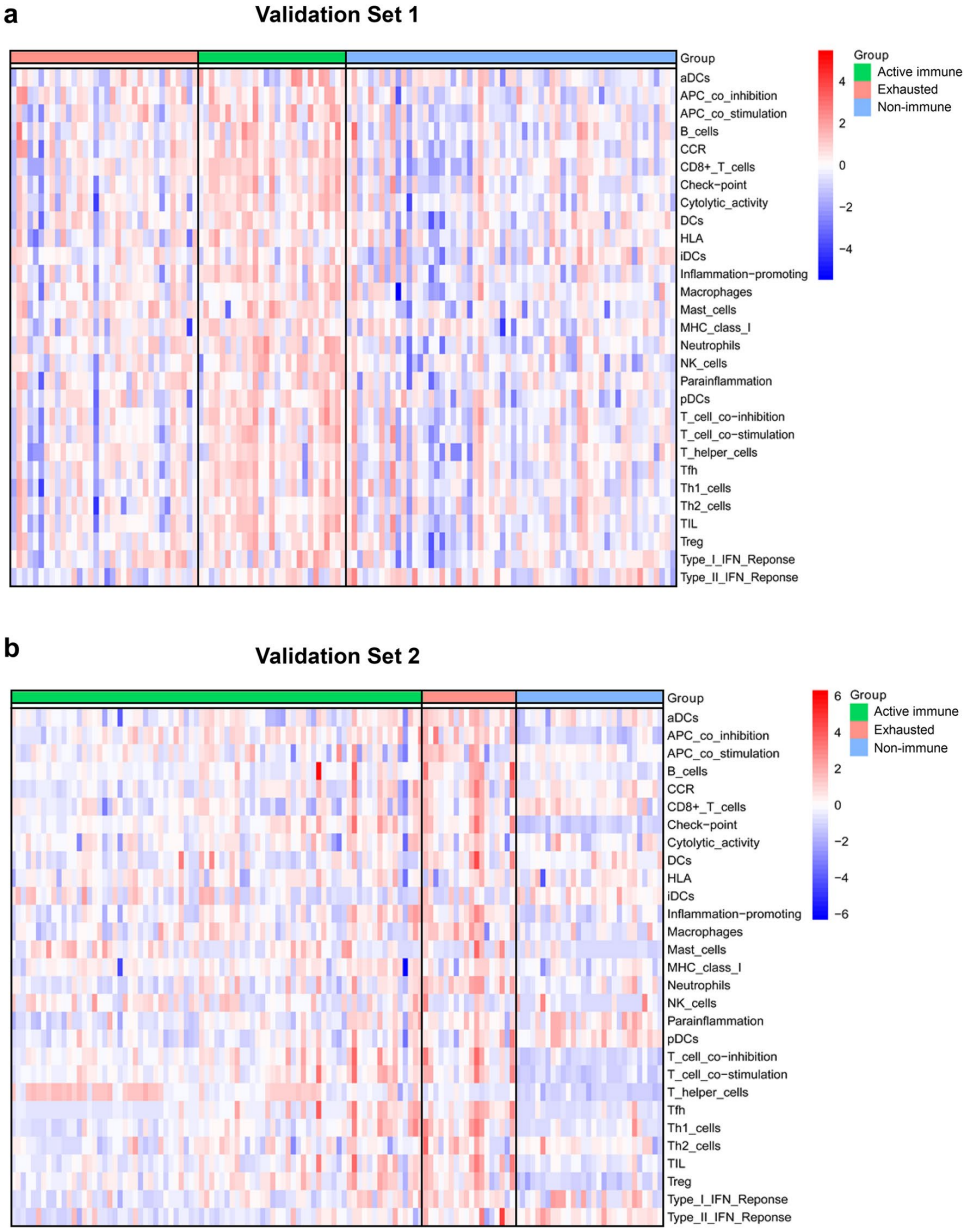
Validation of the immune class in independent publicly available datasets. Presence and molecular characteristics of the immune classes were successfully validated in 2 additional independent datasets. Results in validation set 1 (**a**) and validation set 2 (**b**) are here reported. High and low gene set enrichment scores are represented in red and blue, respectively

The presence of the immune class was further evaluated in 2 additional datasets [14, 15] (GSE63514 and GSE68339) (n = 249, Additional file 1: Fig. S1) using the “NMF method”. Similar to our training cohort, the validation cohort had 108 (43.37%) samples that were successfully predicted to fall within the active immune subgroup, while 52 (20.88%) samples were in exhausted subgroup (Fig. 2). Molecular characterization of the immune class confirmed a significant enrichment of signatures identifying immune cells (i.e., NK cells, B cells, CD8 T cells, mast cells, and macrophages, ANOVA, P < 0.05) and immune-related pathways (i.e., interferon gamma response, interferon alpha response, TGF-β signalling, and TNF alpha signalling via NF-KB, P < 0.05, Additional file 1: Tables S7, S8).

### Correlation of DNA damage with the immune class

**Fig. 3:**
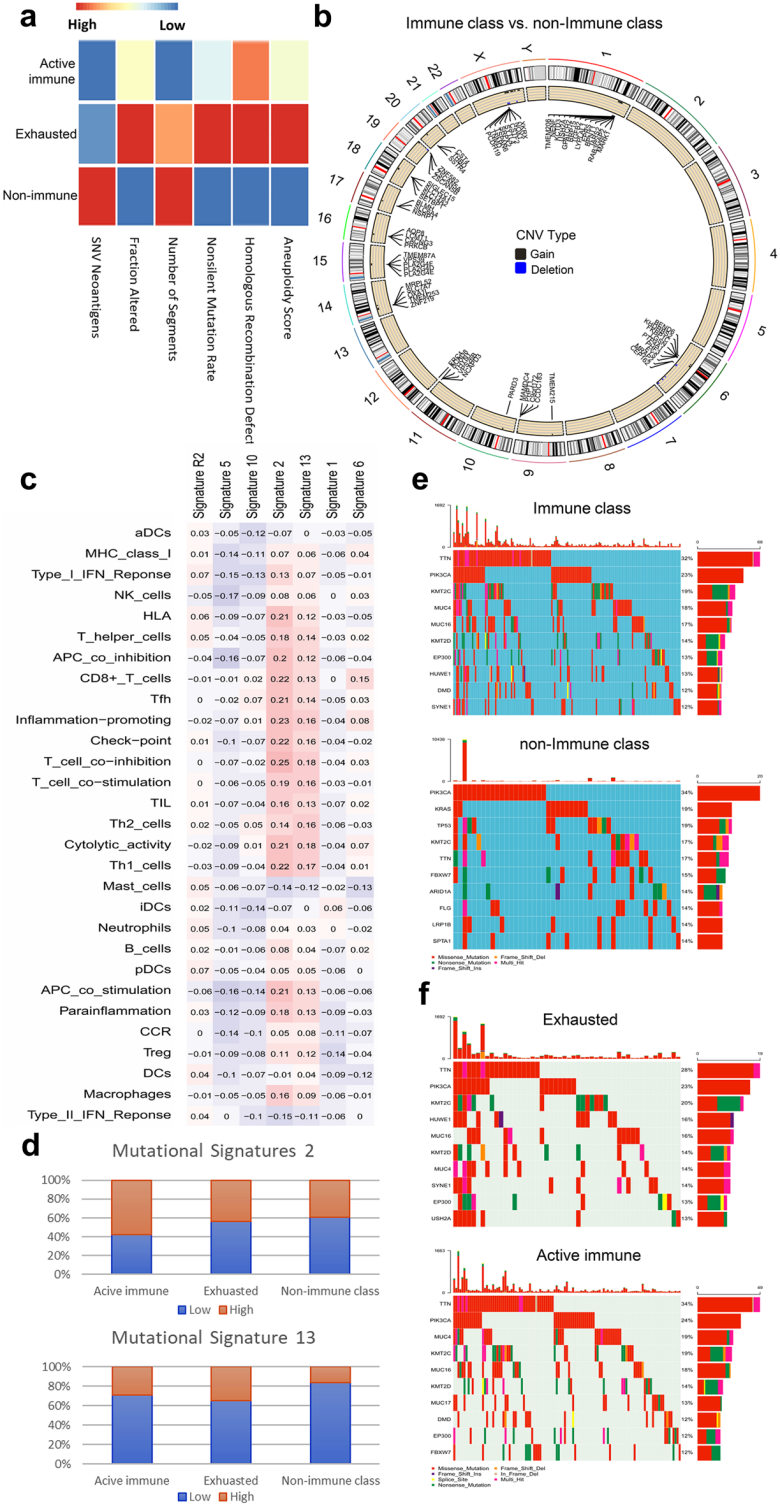
Differences in the mutational landscape according to immune classes. **a** Correlation of DNA damage (rows) with immune subgroups. **b** Differential CNVs between the immune class and non-immune class. Circle: Differential CNV-associated genes in samples according to their chromosomal location. Genes that were gained are labelled in black, and genes that were deleted are labelled in blue. **c** Correlation of immunological parameters with mutational signatures. **d** Correlation between immune classes and MutSigs 2 and 13. **e** OncoPrint of the distribution of mutations in genes between patients of the immune class and non-immune class. **f** OncoPrint of the distribution of mutations in genes between patients of the exhausted class and active immune class

The immune infiltrate was related to measures of DNA damage, including aneuploidy, homologous recombination deficiency (HRD), nonsilent mutation rate, copy number variation (CNV) burden (both in terms of number of segments and fraction of genome alterations) and SNV neoantigens (Fig. 3a). We observed that the exhausted subgroup was associated with the highest aneuploidy, homologous recombination deficiency (HRD), nonsilent mutation rate and fraction of genome alterations (ANOVA and LSD test, P < 0.001). Interestingly, the non-immune class exhibited the highest number of segments and SNV neoantigens (ANOVA and LSD test, P < 0.001). These results may suggest the different effects of multiple smaller copy number events versus larger events on immune infiltration in various immune subgroups. LF correlated negatively with the number of CNV segments, with a significant correlation in the active immune (Spearman correlation, r = − 0.306, P < 0.001) and exhausted (Spearman correlation, r = − 0.384, P = 0.001) subgroups and a non-significant correlation in the non-immune class (Spearman correlation, P = 0.597).

### Correlation of copy number variants with the immune class

Considering the upregulation of immune-related genes in the immune class, we wondered if such regulation could mirror epigenetic alterations in these tumours. According to supervised analysis of level-3 CNV data and somatic or germline mutation data, CNVs have a critical role in cancer development and progression. A chromosomal segment can be deleted or amplified as a result of genomic rearrangements. The CNVs in the immune class compared with the non-immune class are presented in Fig. 3b. The results revealed 380 genes with the trend of differential copy number alterations in the immune class compared with the non-immune class but were not statistically different (P < 0.05, adjP > 0.05) (Additional file 1: Table S9). In addition, 263 genes with differential copy number alterations were in the exhausted subgroup compared with the active immune subgroup but without statistically different (P < 0.05, adjP > 0.05) (Additional file 1: Table S10). Pathway enrichment in the immune class compared with the non-immune class was carried out for both the amplified genes and the deleted genes (Additional file 1: Table S11). The deleted genes were highly associated with pathways related to metabolism, such as the GnRH signalling pathway and ovarian steroidogenesis. The pathways enriched using the amplified gene sets were associated with pathways related to the immune system, such as the pathway involved in Herpes simplex virus 1 infection.

### Correlation of somatic mutations with the immune class

Somatic mutation can be decomposed into mutational signatures (MutSigs). Specific MutSigs have been reported to be associated with specific biological processes including exogenous mutagens (e.g. UV-light, smoking), age-related deamination, and DNA repair machinery [30]. Michael R. Stratton reported a mathematical approach and computational framework to extract mutational signatures from catalogues of somatic mutations from cancers [23]. Correlation analysis of immune parameters and 7 MutSigs was performed. Consistent with previous study, MutSigs 2 and 13 were positively related to immune parameters and displayed a ‘hot’ immune contexture (Fig. 3c). In addition, correlations of immune parameters with MutSig 2 were stronger than with MutSig13. We further explore the correlation between immune classes and MutSigs. MutSigs 2 showed a trend to enriched in active immune subgroup (Chi-square, 57.5%, P = 0.079), while MutSigs 13 exhibited a trend to enriched in exhausted subgroup (Chi-square, 34.8%, P = 0.130) (Fig. 3d).

We further analysed the related mutated genes among subgroups of the cohort (Fig. 3e, f). Analysis of 272 mutation annotated files highlighted 23 highly mutated genes in the non-immune class and 2 highly mutated genes in the immune class but without statistically different (P < 0.05, adjP > 0.05) (Additional file 1: Table S12). Furthermore, we identified a higher relative frequency for 23 mutated genes and a lower relative frequency for 3 mutated genes in the exhausted subgroup compared to the active immune subgroup but were not statistically different (P < 0.05, adjP > 0.05) (Additional file 1: Table S13). Including related mutated genes among subgroups (P < 0.01), SNPs data were correlated with transcriptomic data. We identified KRAS, ITGAX and MCF2 mutation were significant for up-regulation of gene expression at the transcriptive levels (Additional file 1: Fig. S7). KRAS mutation status, which was highly mutated in the non-immune class, was connected to gene expression changes and down-regulation of signaling pathways associating to immune microenvironment (Additional file 1: Tables S14, S15) [32]. Gene ontology identified O-glycan processing and epithelial cilium movement involved in extracellular fluid movement in up-regulation genes, while immune system process and innate immune response pathways in down-regulation genes.

### Correlation of microRNA expression with the immune class

Next, we identified 39 differentially expressed functional miRNAs (DEFMs) (− 1 > log2 FC > 1, FDR < 0.05) between the immune class and non-immune class (Additional file 1: Table S16). Target gene prediction for the miRNAs revealed 95 DEFM-mRNA links in the immune class (Additional file 1: Fig. S7). For miRNAs, 15 DEFM-DEG links were identified, of which 6 were upregulated DEGs in the immune class. However, we failed to identify DEFMs between the exhausted and active immune subgroups.

### Correlation of long noncoding RNA expression with the immune class

**Fig. 4:**
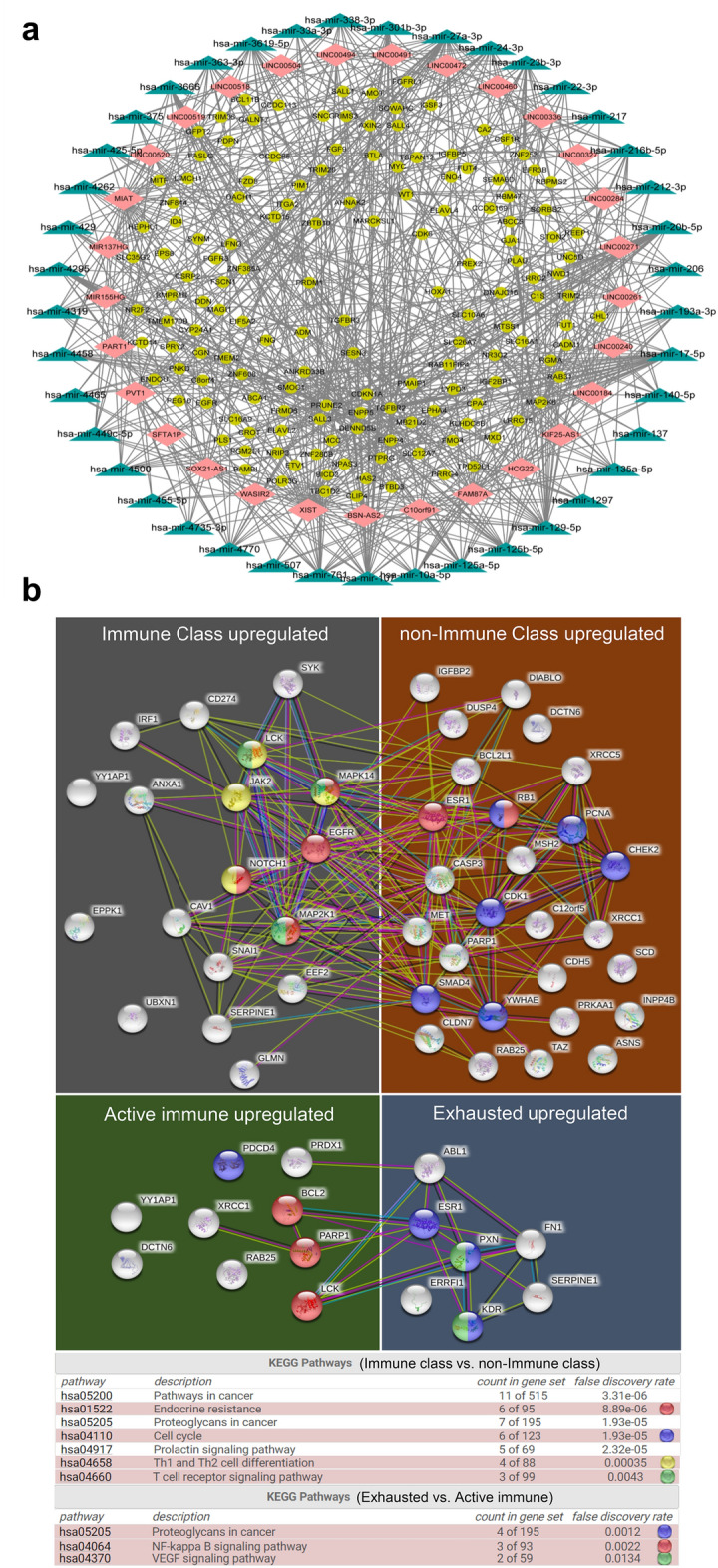
Differences in lncRNA expression and protein expression according to immune classes. **a** The network summarizes complex connections between differentially expressed lncRNAs (pink dots), miRNAs targeted by lncRNAs (green dots), and DEGs (yellow dots, log2 FC > 1 & FDR < 0.05). **b** The network of protein–protein interactions according to the STRING database with highlighted key nodes and key pathways

We investigated 507 differentially expressed lncRNAs (− 1 > log2 FC > 1, FDR < 0.05) between the immune class and non-immune class (Additional file 1: Table S17). Target miRNA prediction revealed 1748 lncRNA-miR links, including 30 lncRNAs and 42/207 miRNAs as part of highly conserved miR families according to the miRcode database, of which 147 of the target mRNAs (n = 1500) were in DEGs and 74/147 DEGs were upregulated in the immune class. Furthermore, between the exhausted and active immune subgroups, we identified 54 differentially expressed lncRNAs and predicted 125 target miRNAs (Additional file 1: Table S18). 23 target mRNAs (n = 1162) were among the DEGs in the exhausted subgroup. Based on the differentially expressed lncRNAs, predicted miRNA links and targeted genes, a complex network was generated that summarizes the underlying molecular traits of distinct tumour immune phenotypes (Fig. 4a, Additional file 1: Fig. S8).

### Correlation of protein expression with the immune class

To explore the immune response at the protein expression level, we investigated the differentially expressed proteins among immune subgroups. Of all 220 measured modified proteins and native proteins, 18/45 proteins had significantly higher levels in the immune class (Additional file 1: Table S19). Furthermore, 9/16 proteins were upregulated in the exhausted subgroup compared to the active immune subgroup (Additional file 1: Table S20). The analysis of protein–protein interactions according to the STRING database highlighted EGFR and ESR1 as key nodes and endocrine resistance, Th1 and Th2 cell differentiation and proteoglycans in cancer as key pathways within the network (Fig. 4b).

### Multi-omics data analyses of genetic and epigenetic regulation according to immune class

**Fig. 5:**
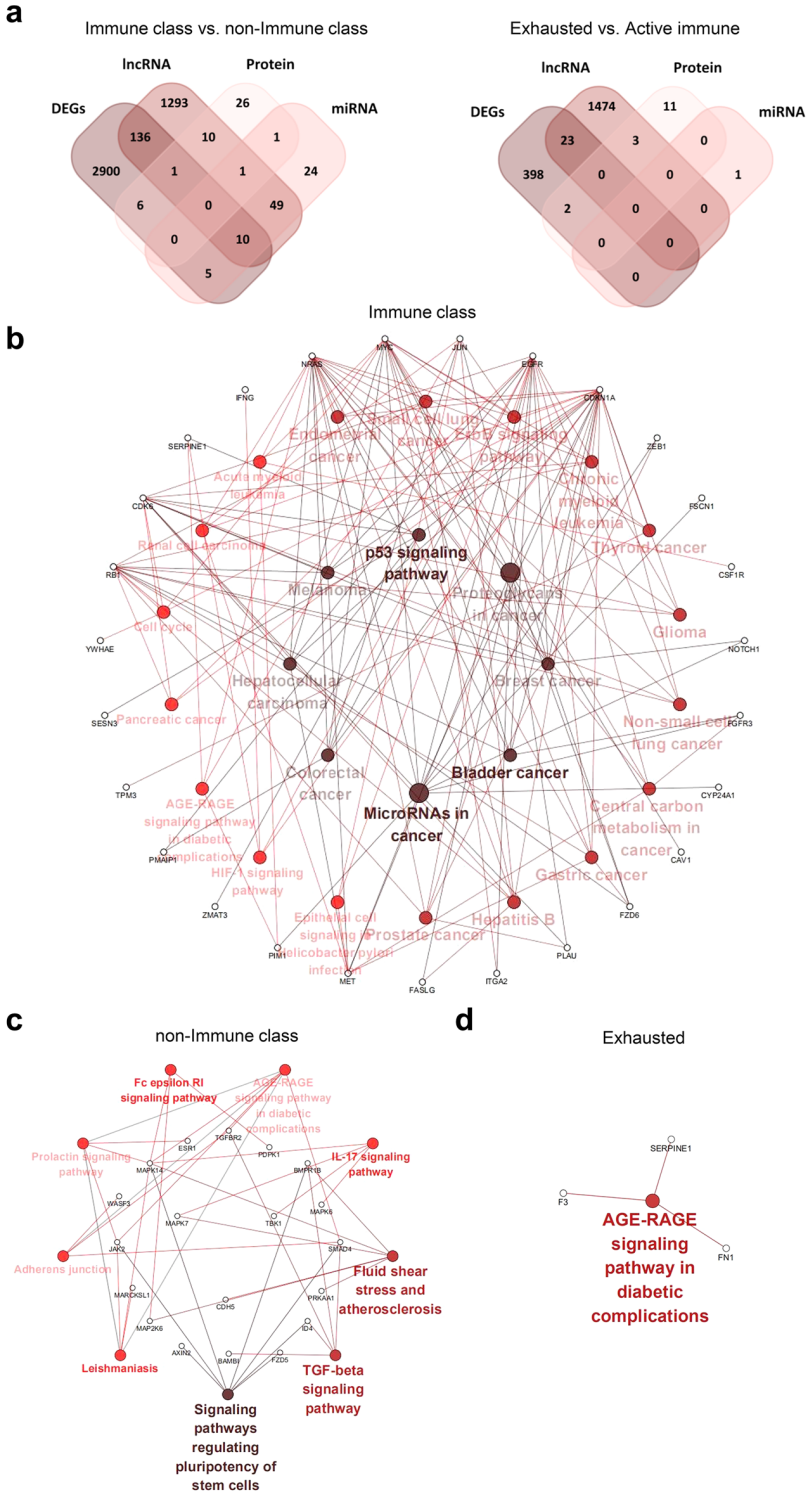

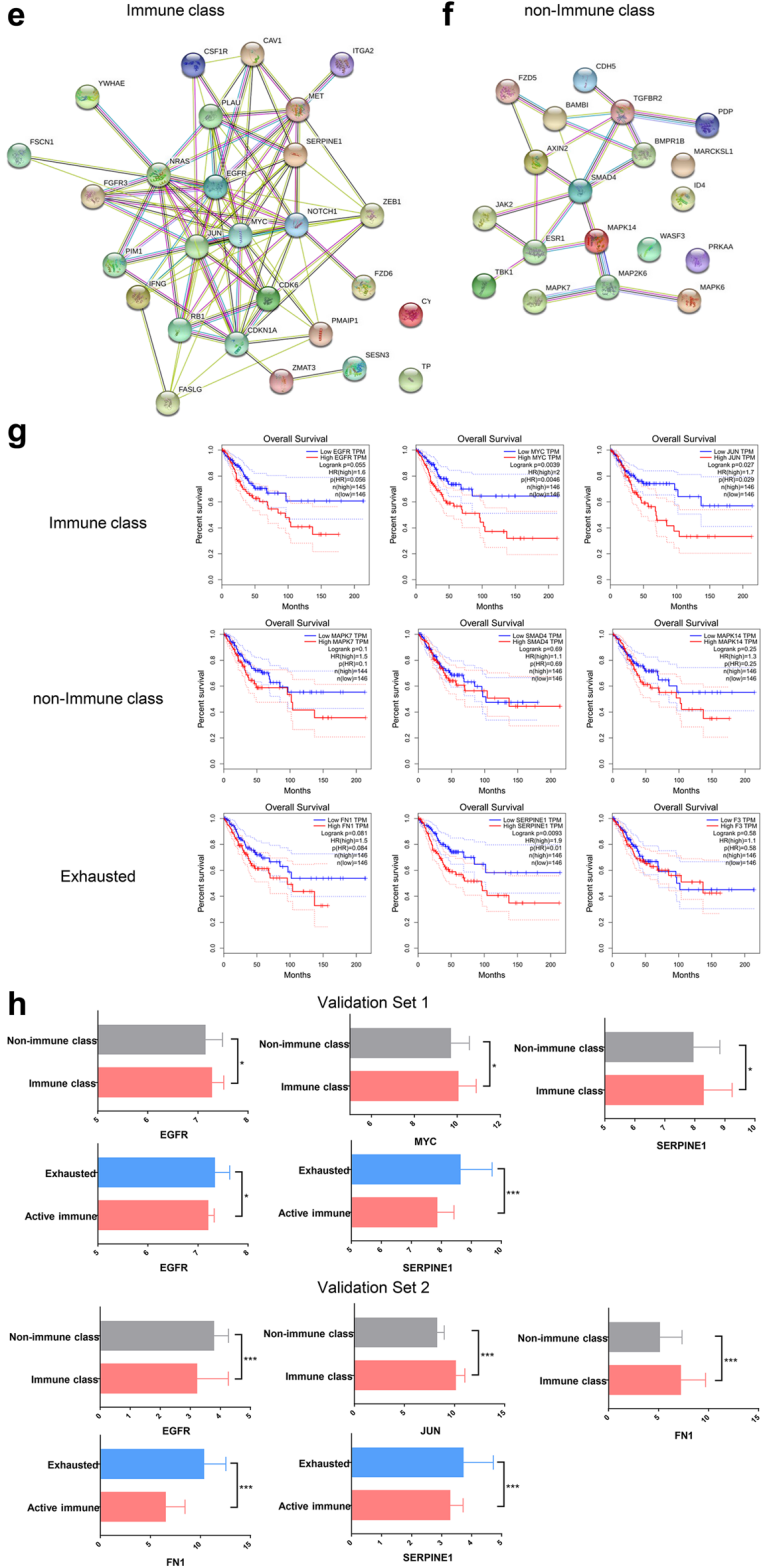
An integrative analysis of multiomics analyses and prognostic impacts according to immune classes. **a** Venn diagrams show different genes between the immune class and the non-immune class (left) or the exhausted class and the active immune class (right) affected by at least one of the indicated CNV, DEG, lncRNA, miRNA, protein or SNP events. The KEGG pathway network was constructed using the most significantly enriched pathways (p < 0.05) in the immune class (**b**), non-immune class (**c**), and exhausted and active immune subgroups (**d**). **e**, **f** The network of protein–protein interactions was constructed using the key genes in the KEGG pathway network in the immune class and non-immune class. **g** The Kaplan–Meier survival curves, including all the key nodes within the protein–protein interaction network, showed that 5 mRNAs were significant for predicting patient overall survival in the immune class, non-immune class and exhausted class. **h** 5 mRNAs expression was evident in 2 validation datasets. *p < 0.05; **p < 0.01; ***p < 0.001

In total, 219 genes were identified in at least two out of the four analyses described above between the immune class and non-immune class. Furthermore, 28 genes were identified between the exhausted subgroup and the active immune subgroup (Fig. 5a). The KEGG pathway network was constructed using the most significantly enriched pathways (p < 0.05) (Fig. 5b–d), which highlighted pathways related to tumourgenesis, cancer proliferation and inflammatory reaction, including p53 signaling pathway, cell cycle, Hepatitis B and epithelial cell signaling in helicobacter pylori infection pathways within the immune class network. The genes enriched in non-immune class were highly associated with pathways related to stem cells and suppression of inflammation, such as the signalling pathways regulating pluripotency of stem cells and TGF-beta signalling pathway. A protein–protein interaction network was constructed using the key genes in the KEGG pathway network (Fig. 5e, f). Considering all the key nodes within the protein–protein interaction network, Kaplan–Meier survival curves showed that EGFR, JUN, MYC, FN1 and SERPINE1 were significant for predicting patients’ overall survival in the subgroups (Fig. 5g), which may provide targets for treatment to improve the prognosis in specific subgroups. Significantly higher EGFR, JUN and MYC expression in immune class as compared to non-immune class was evident in 2 validation datasets. Significantly higher FN1 and SERPINE1 expression in exhausted group as compared to active immune group was also detected in 2 validation datasets (ANOVA, P < 0.05) (Fig. 5h).

### Prognostic association and therapeutic strategies according to immune class

**Fig. 6:**
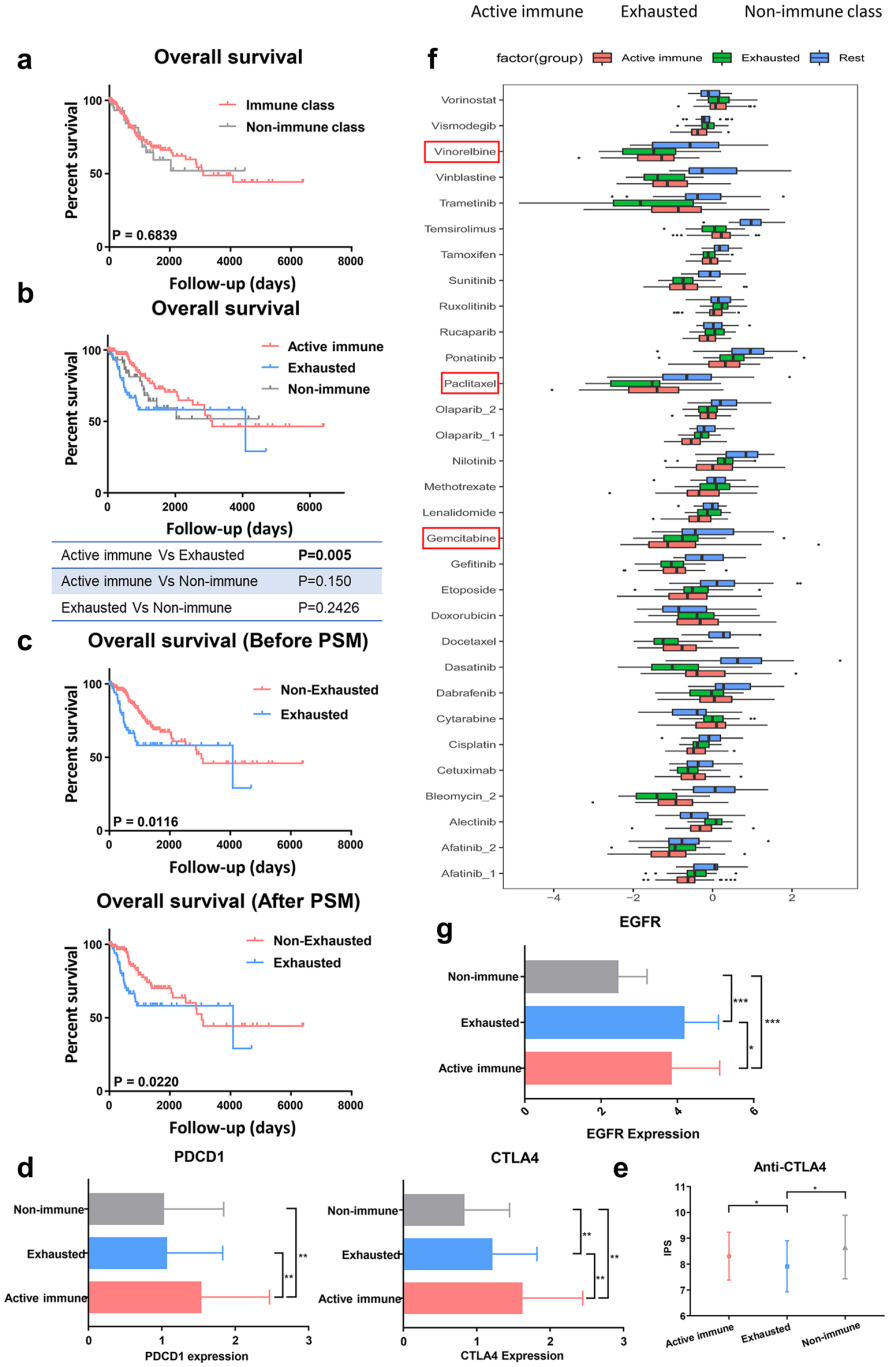
Prognosis and therapeutic strategies according to immune classes. **a** Kaplan–Meier plots of overall survival according to the immune and non-immune classes. **b** Kaplan–Meier plots of overall survival according to the active immune, exhausted, and non-immune classes. **c** Kaplan–Meier plots of overall survival according to the exhausted and non-exhausted classes before and after adjustment for risk factors. **d** The expression of PDCD1 and CTLA4 was significantly upregulated in the active immune class compared with the other classes. **e** Immunophenoscore (IPS) of patients under anti-CTLA4 treatment in the active immune, exhausted, and non-immune classes. **f** Chemosensitivity according to the active immune, exhausted, and non-immune classes. The drugs with red box are used for First- and second-line treatment in CCs. **g** The expression of EGFR was significantly upregulated in the exhausted class compared with the other classes. *p < 0.05; **p < 0.01; ***p < 0.001

We further explored the prognostic implications of the type of immune response by correlating the subgroups with clinicopathologic parameters (Fig. 6a, b). Of note, patients within the exhausted subgroup showed worse overall survival than the remaining patients before and after adjustment for risk factors (log-rank test, P = 0.0116, P = 0.0220) (Fig. 6c). In line with progression-free intervals and disease-specific survival were also the worse in the exhausted subgroup after adjustment for risk factors (Additional file 1: Fig. S9).

Recently, it has been suggested that the abundance of PD-1 mRNA in tumour samples might explain the differences in overall response rates (ORRs) observed following anti-PD-1 monotherapy across cancer types [33]. In our study, PD1 mRNA was significantly enriched in the active immune subgroup (ANOVA and LSD test, P < 0.0001), which suggested a higher response following anti-PD1 monotherapy. Additionally, in the active immune subgroup, CTLA4 mRNA expression was increased with the highest significance (ANOVA and LSD test, P < 0.0001) (Fig. 6d). Charoentong et al. [34] proposed an IPS for defining patients likely to respond to anti-CTLA4 therapy. As illustrated in Fig. 6e, the exhausted subgroup had the lowest IPS under anti-CTLA4 treatment and predicted a poor response to anti-CTLA4 treatment. Recently, stromal immunotypes were proven to be a practical predictive tool to identify patients who would benefit from chemotherapy [35]. We next estimated the chemosensitivity in subgroups, and log-transformed IC50 values are shown in Fig. 6f. Most drugs showed the highest sensitivities in the active immune and exhausted groups (Additional file 1: Table S21). As the hallmarks of the immune class, EGFR was significantly upregulated in the exhausted subgroup compared with the other subgroups, which confirmed the reduced effect of immunotherapy in the exhausted subgroup (Fig. 6g). Interestingly, significantly higher EGFR expression in exhausted group as compared to other groups was also detected in validation set 1 (Fig. 5h).

## Discussion

Despite the promising anticancer activity offered by PD-1 and PD-L1 inhibitors, only a fraction of patients exhibited dramatic responses to single-agent anti-PD-L1/PD-1 antibody treatment, and the objective response rate (ORR) is only approximately 12.2% [5]. This finding highlights the need to identify ideal subgroups for immunotherapy, seek combined therapeutic targets in various immune subgroups to improve the response rate to a single immunotherapy, and make treatment decisions on a personalized basis.

Our study conducted an integrative analysis of multiomics data of the CC tumour immune landscape. We identified 8 key modulators of the tumour immune microenvironment and potential drug targets.

We performed NMF in the training cohort (n = 293). The result identified a class, herein named the immune class. In addition, the robustness of this classification was supported by successful replication in 2 independent datasets. Approximately 78% of samples were classified into the immune class, whose molecular characteristics, including enrichment of immune cell infiltration and enhanced cytolytic activity and type I IFN response, are likely to induce potent clinical responses to immunotherapy [33, 36, 37]. In 2017, approximately 25% of hepatocellular carcinoma (HCC) samples were reported to be classified within immune classes [38]. Consistently, Chen et al. [39] reported that 40% of samples were in immune classes in head and neck squamous cell carcinoma (HNSCC). Our study differs from other recently published studies [38, 39], as we performed an integrative analysis of multiomics data to identify associations between tumour immune classifications, tumour genetics and therapy.

However, the presence of the immune class alone does not absolutely predict response to immunotherapies. It is generally accepted that differences in the effects of immunotherapy are attributed to the heterogeneity of the TME. Therefore, we next identified two microenvironment-based clusters within samples of the immune class, recognized as two separate classes: active immune and exhausted. Although the active immune subgroup was characterized by antitumour characteristics, the exhausted subgroup showed tumour-promoting characteristics (e.g., wound healing signature, enhanced M2 macrophages and absence of CD8 T cells). In particular, the TGF-β response signature, the immunoregulatory cytokine pathway frequently overexpressed in aggressive tumours and angiogenesis, EMT and metastasis signatures were significantly enriched in the exhausted subgroup.

As expected, patients within the active immune subgroup had a significantly favourable prognosis. Integration of copy number, methylation, mRNA and miRNA data using iCluster R package, the cancer genome atlas research network highlighted a pathological pattern-related classification of cervical carcinomas including: a squamous cluster with high expression of keratin gene family members (keratin-high), another squamous cluster with lower expression of keratin genes (keratin-low), and an adenocarcinoma-rich cluster with CpG island hypermethylated (adenocarcinoma) [17]. Integration of immune molecular subgroups with the published pathological pattern-related classification revealed that the active immune subgroup was more common in squamous cluster than in adenocarcinomas. In a study by Heeren, the rate of PD-L1 positivity in patients with SCC was significantly higher than that in patients with AC [40], which suggested higher responses to PD-1 antibodies in active immune subgroup patients. Recently, across 33 diverse cancer types, the Pan-Cancer Atlas of TCGA identified six pan-cancer immune subtypes: wound healing, IFN-γ dominant, inflammatory, lymphocyte depleted, immunologically quiet, and TGF-β dominant [18]. When we integrated six pan-cancer immune subtypes in our study, we observed that most CCs belonged to the wound healing (26%) or IFN-γ dominant (71%) subtypes, while the other four subtypes only accounted for 3% of CCs. Thus, it seems that this classification may not be applicable to CCs. Based on the mRNA expression of the 31-gene radiosensitivity signature and PD-L1, we previously identified the radiosensitivity-PD-L1 classification and predicted the respond to PD-1 immunotherapy and radiotherapy [19]. Interestingly, for the radiosensitivity-PD-L1 classification used in our previous study, the exhausted subgroup was significantly enriched in the RR-PD-L1-high subtype characterized by resistance to radiotherapy or immunotherapy alone and may benefit from combination therapy.

The immune response is more likely regulated by a combination of both extrinsic immune cell infiltration present in the microenvironment and tumour-intrinsic factors based on the genetic composition of the tumour. We observed that the exhausted subgroup was associated with the highest aneuploidy, homologous recombination deficiency (HRD), and nonsilent mutation rates and fraction of genome alterations. Our findings confirmed previous work showing that aneuploidy and fraction of genome alterations (above 0.1 or below—0.1 in the log2-normalized relative copy number (CN) space), which defined as larger copy number events, may play a role in regulating immune evasion and reducing the response to immunotherapy [41, 42], while the number of segments (total number of segments in each copy number profile) was not associated with the exhausted subgroup. Applying an integrative analysis of multiomics data, and taking into account genetic and epigenetic alterations and their impact on differential gene expression, we identified upregulated EGFR and JUN expression as the hallmarks of the immune class. Activated EGFR was reported to induce keratin 5 and keratin 14 expression and was associated with the keratin expression percentages in the immune class, while the non-immune class was mostly composed of adenocarcinomas [43]. Consistently, Noriyasu Hirasawa et al. demonstrated that EGFR transactivation is induced by TNF-α expression in human keratinocytes [44]. Given the higher level of EGFR in squamous cell carcinoma comparing with adenocarcinomas which made up the bulk of the non-immune class, EGFR was significantly enriched in immune class comparing with non-immune class. In immune class, EGFR was significantly enriched in exhausted subgroup comparing with active immune subgroup. Rizvi H et al. reported that reduced response of immunotherapy in patients with EGFR mutations, in particular lung cancer [42]. Our findings confirmed previous work showing that highest level of EGFR expression in exhausted subgroup which was characterized with the worse effect of immunotherapy. Consistently, Esra A Akbay et al. identified a correlation between EGFR pathway activation and a signature of immunosuppression manifested by decreasing CTLs and increasing markers of T-cell exhaustion [30]. The transcription factor JUN was reported to activate the transcription of the promoters of several key UPR effectors, such as XBP1 and ATF4, to inhibit tumour cell apoptosis [45, 46]. The hallmark of the exhausted subgroup, SERPINE1, induced by TGF-β in the microenvironment, may act as a target to reverse the exhausted immune response [47].

Interestingly, the p53 signaling pathway was the most significantly enriched within the immune class. In line with us, Blagih et al. reported that activation of p53 pathway improved endogenous antigen presentation and increased the secretion of IL-6, IL-8 and CCL22, which can regulate the recruitment of leukocytes including macrophages, neutrophils and T cells [48].

Consistently, patients in the active immune subgroup displaying a type I immune response had a favourable prognosis [49]. In addition, in our study, PD-1 mRNA was significantly enriched in the active immune subgroup (p < 0.0001), which suggested a higher response following anti-PD-1 monotherapy. The exhausted subgroup showed the highest proliferation signature and TGF-β response signature, which are frequently overexpressed in aggressive tumours. This may partially explain the poor prognosis in patients within the exhausted subgroup. The exhausted subgroup had the lowest IPS under anti-CTLA4 treatment and had a poor response to anti-CTLA4 treatment. Patients within the exhausted subgroup could benefit from the combination of TGF-β inhibition plus immune checkpoint blockade (NCT02423343 ongoing). In particular, SERPINE1 acted as both a downstream molecule of TGF-β and a key gene in the exhausted subgroup, which may provide an extra target for combination therapy. In addition, several chemotherapeutic drugs may be effective in the exhausted subgroup and need further clinical trials for clinical applications. We found that patients within the active immune subgroup were the most sensitive to cisplatin and were recommended to receive CCRT. One explanation for this result is the enhancement of NK cells induced by platinum compounds [35].

There are some limitations to use TCGA data. For TCGA data, samples with no more than 60% tumour cell nuclei were excluded, thus potentially removing the most immune-infiltrated tumours from our analysis. The degree to which this biases the results is difficult to ascertain.

## Conclusions

**Fig. 7:**
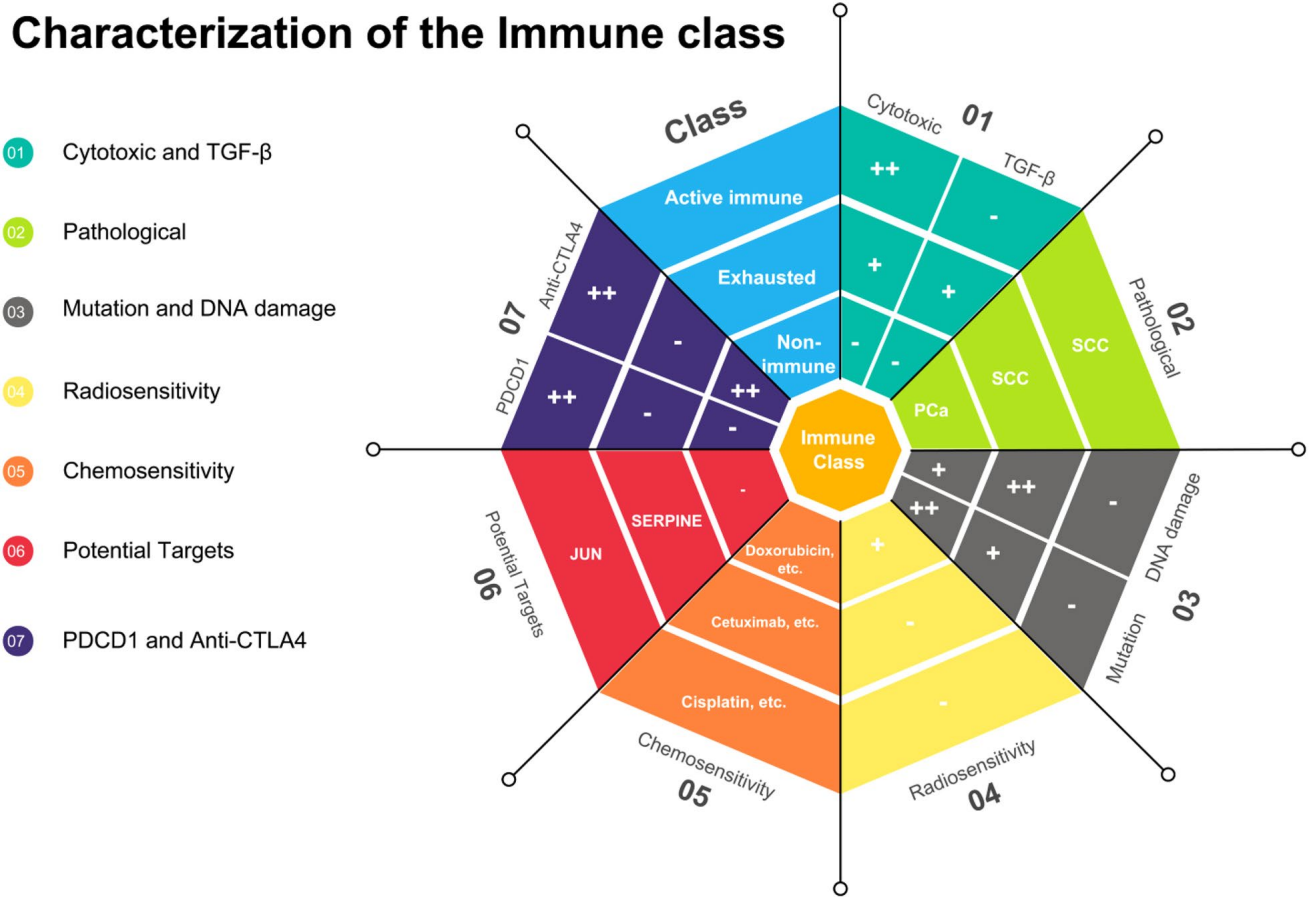
Characterization of the immune class in cervical cancer

Stable and reproducible immune subgroups were found and were associated with immune modulatory alterations, genetic and epigenetic events, prognosis and response to various therapeutic strategies and might help predict ideal candidates to receive specific combination therapies (Fig. 7). Further investigations of this immune classification in a large cohort of patients receiving individual treatment are needed to determine its potential use in predicting response in clinical trials.

## Supplementary Information

**Additional file 1.** **Fig. S1**. Flow chart of the study. A total of 542 human cervical cancer samples were analysed in this study. A training cohort including 293 samples was virtually microdissected to identify an Immune class. Validation was then performed in 2 independent datasets. **Fig. S2**. Identification of an immune expression pattern. (A) We used Nonnegative Matrix Factorization (NMF, k=3 factors) to analyze the microarray-based gene expression data of 293 samples. One of the 3 expression factors showed the lowest intra-tumoral immune cells (indicated in blue) as shown in the heatmap. High and low gene set enrichment scores are represented in red and blue, respectively. **Fig. S3**. Identification of the immune class. Heatmap indicates NMF consensus-clustering on exemplar genes and the Immune class. High and low gene set enrichment scores are represented in red and blue, respectively. The tumor purity, ESTIMATES score, immune enrichment score and stromal enrichment score is also indicated. **Fig. S4**. Molecular characterization of the immune class. Gene set enrichment analysis (GSEA) between the immune class and non-immune class confirmed enrichment of inflammation–related pathways, signatures of immune cells (p<0.05, FDR<0.05). **Fig. S5**. Molecular characterization of the two subtypes of the tumour microenvironment in the immune class: active immune and exhausted classes. Gene set enrichment analysis (GSEA) between the exhausted class and active immune subgroups (p<0.05, FDR<0.05). **Fig. S6**. Correlation between SNP data and transcriptomic data. KRAS, ITGAX and MCF2 mutation were significant for up-regulation of gene expression (p<0.05). **Fig. S7**. Differentially expressed miRNAs between immune class and non-immune class. The network summarizes complex connections between differentially expressed miRNAs (green dots) and targeted gene (yellow dots) (-1>log2 FC>1, FDR < 0.05). **Fig. S8**. Differentially expressed lncRNAs between exhausted and active immune subgroups. The network summarizes complex connections between differentially expressed lncRNAs (pink dots), targeted miRNAs (green dots) and targeted gene (yellow dots) (-1>log2 FC>1, FDR < 0.05). **Fig. S9**. Disease-specific survival and progression-free intervals according to immune classes before and after adjustment for risk factors. Disease-specific survival and progression-free intervals were the worse in the exhausted subgroup before and after PSM. **Table S1** DEGs (Immune-vs-non-immune). **Table S2** GSEA (Non-immune-vs-Immune). **Table S3** Univariate analysis for overall survival (OS), disease-specific survival (DSS) and progression-free interval (PFI). **Table S4** Multivariate analysis for for overall survival (OS), disease-specific survival (DSS) and progression-free interval (PFI). **Table S5** DEGs (Exhausted-vs-Active immune). **Table S6** GSEA (Exhausted-vs-Active immune). **Table S7** GSEA Validation set 1 (Non-immune-vs-Immune). **Table S8** GSEA Validation set 2 (Nonimmune-vs-Immune). **Table S9** CNV (Immune vs Non-immune). **Table S10** CNV (Exhausted vs Active immune). **Table S11** The pathway enrichment of genes involving in the amplification and deletion (Immune vs Non-immune). **Table S12** SNP (Immune vs Non-immune). **Table S13** SNP (Exhausted vs Active immune). **Table S14** connect KRAS mutation status to gene ontology - Biological process (upregulation). **Table S15** connect KRAS mutation status to gene ontology - Biological process (downregulation). **Table S16** DEFM (Immune vs Non-immune). **Table S17** Differentially expression lncRNAs (Immune vs Non-immune). **Table S18** Differentially expression lncRNAs (Exhausted vs Active immune). **Table S19** Differentially expressed proteins (Immune vs Non-immune). **Table S20** Differentially expressed proteins (Active immune vs Exhausted). **Table S21** Sensitive chemotherapeutic drugs in subgroups.

## Data Availability

The datasets generated and/or analysed during the current study are available in the Genomic Data Commons Data Portal (https://portal.gdc.cancer.gov/), UCSC Xena platform (https://genome-cancer.ucsc.edu/) and the NCBI Gene Expression Omnibus (GSE63514 and GSE68339).
